# Antineuroinflammatory Effect of *Amburana cearensis* and Its Molecules Coumarin and Amburoside A by Inhibiting the MAPK Signaling Pathway in LPS-Activated BV-2 Microglial Cells

**DOI:** 10.1155/2022/6304087

**Published:** 2022-04-28

**Authors:** Ana Bruna de Araújo, Francisco Vinícius Clemente Serra Azul, Francisca Raysse Mesquita Silva, Talysson Silva de Almeida, João Victor Nunes Oliveira, Antônia Torres Ávila Pimenta, Antônio Marcos Esmeraldo Bezerra, Nuno J. Machado, Luzia Kalyne Almeida Moreira Leal

**Affiliations:** ^1^Centro de Estudos Farmacêuticos e Cosméticos (CEFAC), Departamento de Farmácia, Faculdade de Farmácia, Odontologia e Enfermagem, Universidade Federal do Ceará, Fortaleza, CE, Brazil; ^2^Programa de Pós-Graduação em Ciências da Saúde, Universidade Federal do Ceará, Sobral, CE, Brazil

## Abstract

Microglia plays an important role in the neuroinflammatory response, identified as one of the major factors in the development and progression of neurodegenerative diseases. *Amburana cearensis* and its bioactive compounds, including coumarin (CM), vanillic acid (VA), and amburoside A (AMB), exert antioxidant, anti-inflammatory, and neuroprotective activities, on 6-OHDA-induced neurotoxicity in rat mesencephalic cells determined by our group. The present study investigated the anti-inflammatory effect of the dry extract from *A. cearensis* (DEAC), CM, AMB, and VA on lipopolysaccharide- (LPS-) stimulated microglial cells and elucidated the possible molecular mechanism of action. The DEAC was characterized by HPLC-PDA (chemical markers: CM, AMB, and VA). The BV-2 microglial cell line was pretreated with increasing concentrations of DEAC, CM, AMB, or VA in the presence or absence of LPS to evaluate the toxicity and anti-inflammatory activity. The cytotoxicity of DEAC, CM, AMB, or VA on BV-2 cells was evaluated by the MTT test, the free radical scavenging activity of test drugs was investigated, and the nitric oxide (NO) production was determined using the Griess reagent, while cytokine levels were measured by ELISA. The expressions of toll-like receptor 4 (TLR-4), nuclear factor kappa B (NF-*κ*B), MAPK members (JNK and ERK1/2), and iNOS were determined through Western blot analysis. DEAC, CM, AMB, or VA (5-100 *μ*g/mL) did not induce any detectable cytotoxicity in BV-2 cells. All test drugs (100 *μ*g/mL) showed free radical scavenging activity (hydroxyl and superoxide radicals); however, only DEAC, CM, and AMB (5-100 *μ*g/mL) significantly reduced NO production. DEAC (100 *μ*g/mL), as well as CM (50 and 100 *μ*g/mL) and AMB (25 *μ*g/mL), reduced at least 50% of NO produced and markedly decrease the production of TNF-*α* and IL-6 but they did not significantly affect IL-10 levels. Only DEAC (100 *μ*g/mL) and AMB (25 *μ*g/mL) reduced the expression of iNOS, and they did not affect arginase activity. DEAC (100 *μ*g/mL) suppressed the activation of the MAPKs JNK and ERK1/2 in LPS-activated BV-2 cells but it did not suppress the expression of TLR-4 nor the phosphorylation of NF-*κ*B. In conclusion, DEAC, CM, and AMB exerted anti-inflammatory activity in LPS-activated microglial cells as observed by the reduction in the production of inflammatory mediators and the expression of iNOS. We identified the MAPK signaling pathway as a probable mechanism of action to the anti-inflammatory effects observed.

## 1. Introduction

There is increasing evidence of the important role of neuroinflammation in the pathogenesis of several neurodegenerative diseases, including Alzheimer's disease and Parkinson's disease [1–3]. The neuroinflammatory process includes a variety of events marked by the accumulation of various proinflammatory mediators, such as tumor necrosis factor alpha (TNF-ɑ), interleukin 1*β* (IL-1*β*), interleukin 6 (IL-6), prostaglandins, nitric oxide (NO) (generated by inducible nitric oxide synthase (iNOS) and reactive oxygen species (ROS)). Microglia and astrocytes are the innate immune cells primarily involved in these proinflammatory mechanisms which cause damage to neuronal networks. In parallel, under physiological conditions, these cells have a protective function in preserving the homeostasis of the central nervous system (CNS) by secreting anti-inflammatory mediators and neurotrophic factors, such as transforming growth factor-*β*, interleukin 10 (IL-10), and arginase-1 [3, 4]. Thus, controlling microglial activation or modulating its phenotypes in response to damage-associated stimuli may be a promising strategy in the development of a therapy for neuroinflammatory diseases.

Microglial cells can be activated by various endogenous and environmental stimuli such as IFN-*γ* (interferon-gamma), glutamate, arachidonate, and lipopolysaccharide (LPS). LPS is found on the outer membrane of Gram-negative bacteria, acting as an endotoxin that binds to specific receptors, and it has been widely used to study inflammation due to its reproducible action and easy handling [5]. LPS induces activation of the toll-like receptor 4 (TLR-4) which activates nuclear factor kappa B (NF-*κ*B) and mitogen-activated protein kinases (MAPKs) that play critical roles in the production of inflammatory mediators [6, 7].

Due to a deficit in therapeutical options, herbal products have been the focus of several studies for the treatment or prevention of neurodegenerative diseases [8, 9]. *Amburana cearensis* A. C. Smith (Fabaceae), a plant native to the Brazilian semiarid region, has been extensively studied by our group that determined its chemical profile and pharmacological properties [10–13]. Other previous studies developed by our group [10, 14–18] allowed us to determine the anti-inflammatory and antioxidant activities of the extract of *A. cearensis* (wild or cultivated) and/or its chemical constituents, such as coumarin (CM), amburoside A (AMB, phenol glucoside), and vanillic acid (VA, phenol acid). Outside the brain, they exert anti-inflammatory activity mainly by inhibiting the accumulation and activation of polymorphonuclear cells and the production of cytokines and ROS. Additionally, in rat mesencephalic cell cultures exposed to 6-hydroxydopamine (6-OHDA), Leal and colleagues [15] showed that AMB reduced the damage caused by this neurotoxin through the inhibition of the increase in lipidic peroxidation and production of nitrite. These data suggest that this phenol glucoside, among the main chemical constituents of *A. cearensis*, could provide benefits as a therapeutic agent in neurodegenerative diseases such as Parkinson's disease. Therefore, the present study was designed to evaluate the anti-inflammatory effects of a standardized dry extract of *A. cearensis* and its chemical constituents (CM, AMB, and VA) in LPS-stimulated microglial cells. Moreover, it is aimed at contributing to the elucidation of the mechanisms of action involved by evaluating the role of the signaling pathways of TLR-4, NF-*κ*B, and MAPKs.

## 2. Materials and Methods

### 2.1. Preparation and Chemical Characterization of Dried Extract from *Amburana cearensis* Extract (DEAC)

To achieve a sustainable alternative for economic utilization of *A. cearensis*, in the last years, our research group became focused also on *A. cearensis* cultivated plant which has chemical and biological properties similar to the wild variety [16]. Seeds of *A. cearensis* were collected at the Quixeramobim region, Ceará State, Brazil, and voucher specimen (n° 52618) was deposited in the herbarium Prisco Bezerra, Department of Biology, Federal University of Ceará. *A. cearensis* seeds were cultivated, and seven months later, the plant was harvested from the garden beds, dried, and ground. The extraction was carried out by maceration for 24 h with ethanol as previously determined by our laboratory [17]. The drying process of the ethanol extract was performed at room temperature using a mini spray-dryer model LM MSD 1.0 (Labmaq do Brazil Ltda, Ribeirão Preto, SP, Brazil). The colloidal silicon dioxide was used as drying carrier (30% of the solid residues) with a feed flow of 1 L/h, inlet temperature of 100°C, and airflow of 40 L/min.

### 2.2. HPLC-PDA Analysis

Dried extract of *A. cearensis* (DEAC) was analyzed by high-performance liquid chromatography–photodiode array (HPLC-PDA) (Waters) according to the method developed previously by our laboratory [10]. The analysis of the three chemical markers (coumarin (CM), amburoside A (AMB), and vanillic acid (VA)) was performed through their calibration curves, obtained by injection of external standards. The analysis of DEAC was performed in a C18 reverse phase column (4.6 mm × 250 mm) at a temperature of 45°C. The mobile phase was composed of acetonitrile (A), 0.01% phosphoric acid (B), and n-propanol (C), flow of 1 mL per minute. Detection was performed in a PDA detector using the wavelengths of 219 nm, 277 nm, and 220 nm for the determination of VA, CM, and AMB, respectively. The HPLC-PDA analysis of the DEAC allowed the identification and quantification of vanillic acid, amburoside, and coumarin.

### 2.3. Chemicals and Antibodies

The amburoside A (AMB) was isolated from *A. cearensis* according to the methodology described previously by our group [18]. Vanillic acid (VA), coumarin (CM), *N*-(1-Naphthyl) ethylenediamine dihydrochloride (NEED), 3-(4,5-dimethylthiazol-2-yl)-2,5-diphenyltetrazolium bromide (MTT), dimethyl sulfoxide (DMSO), bovine serum albumin (BSA), 2-thiobarbituric acid (TBA), nitrotetrazolium blue chloride (NBT), hypoxanthine (HPX) and xanthine oxidase (XOD), phosphoric acid (H_3_PO_4_), ferric chloride (FeCl_3_), hydrogen peroxide (H_2_O_2_), and L-ascorbic acid (vitamin C) were obtained commercially from Sigma-Aldrich, USA. Sulfanilamide and ethylenediaminetetraacetic acid tetrasodium salt dehydrate (EDTA) were purchased from Dinâmica Química Conteporânea, Brazil. Trichloroacetic acid (TCA) was acquired from Êxodo Científica, Brazil.

Fetal bovine serum (FBS) and cell culture medium RPMI-1640 were purchased from Gibco, USA. Murine microglial (BV-2) cells were acquired from Banco de Células do Rio de Janeiro, Brazil.

The antibodies anti-NF-*κ*B, *p*-NF-*κ*B, JNK, *p*-JNK, and *p*-ERK were obtained from Cell Signaling Technologies. TLR-4 antibody was obtained from Invitrogen, Carlsbad, CA, USA. Anti-iNOS, ERK, *β*-actin antibodies, and secondary antibodies (anti-rabbit and anti-mouse) were obtained from Abcam Cambridge, UK. ELISA kits for TNF-*α*, IL-6, IL-10, and IL-1*β* were purchased from BD Bioscience Pharmingen, San Diego, CA, USA.

### 2.4. Cell Culture Conditions

The murine BV-2 cells were maintained in RPMI-1640 medium supplemented with 10% fetal bovine serum. The cells were grown to 70-80% confluency and incubated at 37°C in an atmosphere of 5% CO_2_.

### 2.5. Cell Viability Assay

Before conducting the anti-inflammatory activity test of the DEAC and its chemical constituents, we performed the cytotoxicity test first in BV-2 cells. The MTT test evaluated BV-2 cells' viability using the method previously described by Mosmann [19]. The cells (1 × 10^5^ cells/mL) were plated in 96-well plates and treated with DEAC, CM, VA, or AMB (5, 10, 25, 50, and 100 *μ*g/mL) and 0.1% dimethyl sulfoxide (DMSO, Sigma-Aldrich, USA) (drug vehicle) for 24 h. After the incubation period, the salt 3-(4,5-dimethyl-2-thiazolyl)-2,5-diphenyl-2H-tetrazolium bromide (MTT) solution (0.5 mg/mL final concentration) was added into the plate, then incubated for 90 minutes at 37°C. At last, the cells were centrifuged at 200 g for 10 minutes, the supernatant discarded, and the formazan crystals dissolved in DMSO. The absorbance of each well was recorded at 570 nm by using a plate reader (Bio-Tek, Winooski, VT, USA).

### 2.6. NO Measurement

The concentration of nitrite in LPS-induced BV-2 cells was determined by the Griess assay [20]. The cells (1 × 10^6^ cells/mL) were plated in 96-well plates and incubated with DEAC, CM, VA, or AMB (5, 10, 25, 50, and 100 *μ*g/mL) and 0.1% DMSO (drug vehicle), and 1 h later, LPS (0.5 *μ*g/mL) was added. After 24 h, Griess reagent (1% sulfanilamide in 5% phosphoric acid and 0.1% *N*-(1-Naphthyl) ethylenediamine dihydrochloride (NEED) in distilled water, 1 : 1) was added to cell medium at room temperature, and 15 minutes later, absorbance at 540 nm was measured on a plate reader. The concentrations of NO were calculated from the standard curve generated by known concentrations of sodium nitrite.

### 2.7. Measurement of Hydroxyl Radical

The hydroxyl radical was measured using the deoxyribose method according to Zhao and colleagues [21] with modifications. Ascorbic acid (1 mM), EDTA (1 mM), H_2_O_2_ (10 mM), FeCl_3_ (1 mM), and deoxyribose (DOR) (36 mM) were added to a reaction tube with DEAC (100 *μ*g/mL), CM (100 *μ*g/mL), VA (100 *μ*g/mL), AMB (100 *μ*g/mL), or 0.1% DMSO (drug vehicle) in 25 mM phosphate buffer (pH 7.4) and incubated for 1 h at 37°C. TCA 10% and TBA 1% (prepared in NaOH 50 mM) were added, and the solution was heated in a water bath at 85°C for 15 minutes. Absorbance at 532 nm was measured.

### 2.8. Assay for Superoxide Radical Scavenging Activity

Superoxide generation was measured by a slight modification of the previous method described by Hodgson and Fridovich [22] using the hypoxanthine/xanthine oxidase (Sigma-Aldrich, USA) system. Nitrotetrazolium blue chloride (NBT), hypoxanthine (HPX), and xanthine oxidase (XOD) solutions were prepared in Tris buffer. DEAC, CM, VA, or AMB at 100 *μ*g/mL, or 0.1% DMSO, were added to the reaction solution containing 0.05 M Tris buffer, 5 × 10^−3^ M HPX, and 10^−3^ M NBT. Then, 1.67 *μ*g/mL XOD was added, except in the negative control. The absorbance was measured at 560 nm every minute for 21 min.

### 2.9. Enzyme-Linked Immunosorbent Assay (ELISA)

BV-2 cells were pretreated with DEAC (100 *μ*g/mL), AMB (25 *μ*g/mL), CM (50 and 100 *μ*g/mL), or 0.1% DMSO (drug vehicle) for 1 h, then stimulated with LPS (0.5 *μ*g/mL) for 24 h. The levels of TNF-*α*, IL-6, IL-1*β*, and IL-10 in the culture supernatant were quantified using commercially available ELISA kits (BD Bioscience Pharmingen, San Diego, CA, USA) according to the manufacturer's protocol. The absorbance at 450 nm was measured using a microplate reader.

### 2.10. Western Blot Analysis

BV-2 microglial cells (1 × 10^6^ cells/mL) were pretreated with DEAC (100 *μ*g/mL), CM (50, 100 *μ*g/mL), AMB (25 *μ*g/mL), or 0.1% DMSO (drug vehicle) for 1 h. LPS (0.5 *μ*g/mL) was added, and the cells were incubated for 1 h or 24 h. The cells were harvested, and adherent cells were resuspended in ice-cold phosphate-buffered saline (PBS). Cellular proteins were extracted by incubating with radioimmunoprecipitation assay buffer (RIPA) containing protease inhibitor cocktail (1 : 20), phenylmethylsulfonyl fluoride (1 : 50), and phosphatase inhibitor cocktail set III (1 : 20). The cell lysate was centrifuged at 12500 × g for 10 min at 4°C, and the supernatant was kept as the protein extract. Protein concentration was determined with a BCA kit using bovine serum albumin (BSA) as standard, and the protein concentration of all samples was normalized with *Laemmli* buffer. Equal amounts of protein (30 *μ*g) from each sample were subjected to 7.5-10% SDS-PAGE and transferred to PVDF membranes. After blocking at room temperature with 5% nonfat dry milk or 5% BSA for 1 h, the blocked membranes were incubated for 18 h with primary antibodies against p-NF-*κ*B 1 : 1000 (#3033S), NF-*κ*B 1 : 2000 (#6956S), p-ERK1/2 1 : 1000 (#9101S), ERK1/2 1 : 1000 (ab36991), p-JNK 1 : 1000 (#9255S), JNK 1 : 2000 (#9252S), iNOS 1 : 1000 (ab178945), TLR-4 1 : 200 (482300), and *β*-actin 1 : 5000 (ab8226) at 4°C. After washing three times with TBS-T (10 mM Tris-HCl (pH 7.6), 150 mM NaCl, 0.1% Tween 20), the membranes were incubated with the secondary antibody for 2 h at room temperature. Protein bands were visualized using enhanced chemiluminescence reagent (Bio-Rad, USA) in an imaging system (ChemiDoc MP, Bio-Rad, USA) [23], and the protein levels were analyzed using the ImageLab 6 software (Bio-Rad, USA) and normalized with *β*-actin or total proteins.

### 2.11. Analysis of Arginase Activity

Arginase activity was evaluated according to the method described by Corraliza et al. [24]. BV-2 cells (1 × 10^6^ cells/mL) were pretreated with DEAC or CM (100 *μ*g/mL) or 0.1% DMSO (drug vehicle), for 1 h before the addition of LPS (0.5 *μ*g/mL), or vehicle. After 24 h, cells were lysed with buffer containing 0.1% Triton X-100. The enzyme was activated by heating the plates for 10 minutes at 55°C, using enzyme activation buffer (10 mM MnCl_2_, 25 mM Tris-HCl). The hydrolysis of L-arginine (25 *μ*L, 0.5 M, pH 9.7) was induced through the incubation of lysate (50 *μ*L) with 25 *μ*L of L-arginine (Sigma-Aldrich, USA) 0.5 M (pH 9.7) during 60 min at 37°C. The reaction was stopped with 400 *μ*L of buffer containing H_2_SO_4_/H_3_PO_4_/H_2_O (1/3/7, *v*/*v*/*v*). After the addition of *α*-isonitrosopropiophenone (25 *μ*L, 9%) and heating for 30 minutes at 95°C, the concentration of urea (final product of the reaction) was determined by spectrophotometric analysis (540 nm). The concentrations of urea were interpolated from the linear equation obtained from the standard curve generated by known concentrations of urea (1.5 to 300 *μ*g/mL).

### 2.12. Statistical Analysis

Statistical analyses were performed using the GraphPad Prism 6.01 software (GraphPad Software, Inc.), and all results were expressed in ± SEM (standard error of mean). Differences were evaluated by one-way ANOVA or two-way ANOVA with Bonferroni's post hoc test or one-sample *t*-test. Statistical significance was considered when *p* < 0.05.

## 3. Results

### 3.1. Standardized DEAC and Its Active Compounds Are Not Toxic to BV-2 Microglial Cells

**Figure 1:**
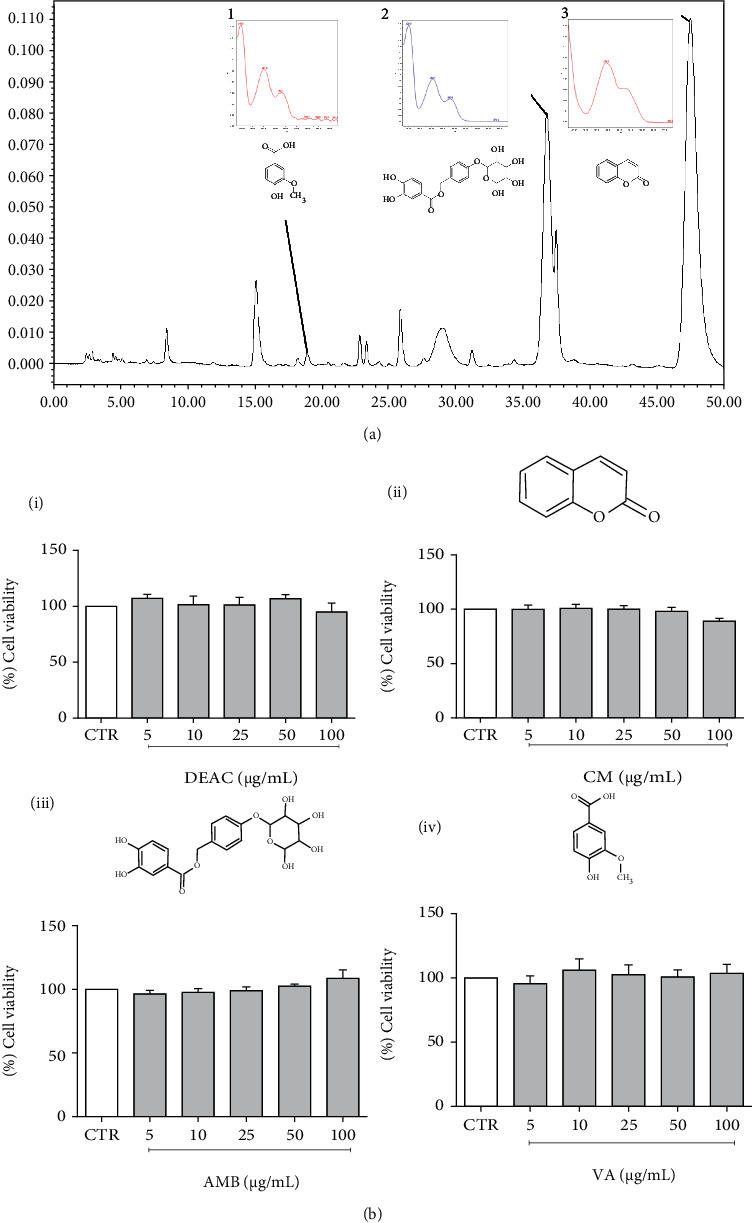
Chromatographic profile of dry extract from *A. cearensis* (DEAC) and identification of three compounds (a). Effect of DEAC (i), CM (ii), AMB (iii), and VA (iv) (5-100 *μ*g/mL) in BV-2 cell viability measured through the MTT assay. Data are shown as percentage of controls (CTR; 0.1% DMSO). Results are expressed as the mean ± SEM. ANOVA test followed by the Bonferroni posttest, *n* = 4/group.

The dried extract of *A. cearensis* (DEAC) was characterized by HPLC-PDA as shown in Figure 1(a). The HPLC analysis allowed to detect and quantify three compounds (mg/g of dried extract): vanillic acid (2.70 ± 0.01 mg/g), amburoside A (30.40 ± 0.01 mg/g), and coumarin (70.07 ± 0.01 mg/g). To first evaluate the effect of DEAC and its chemical constituents on cell viability by MTT test, we pretreated BV-2 microglial cells with increasing concentrations of test drugs. As shown in Figure 1(b), the treatment of cells with DEAC, CM, AMB, or VA (5, 10, 25, 50, and 100 *μ*g/mL) did not significantly affect the viability of cells when compared to the control group (100% of viability) (see Figure 1).

### 3.2. DEAC, CM, and AMB Reduce NO Production in LPS-Stimulated Microglial Cells

**Figure 2:**
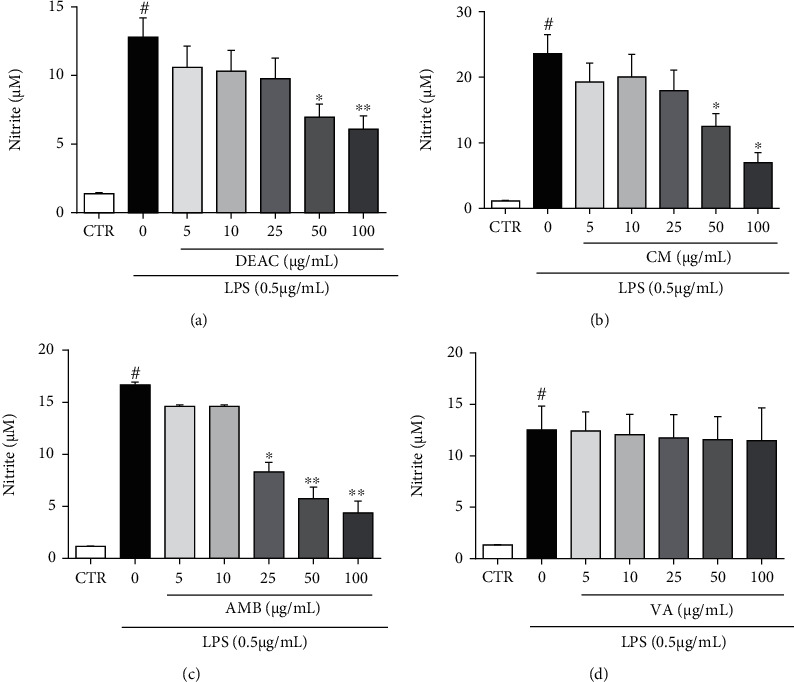
Effect of DEAC (a), CM (b), AMB (c), and VA (d) (5-100 *μ*g/mL) in LPS-induced nitrite production. BV-2 cells were pretreated with the drugs for 1 h and incubated with or without LPS (0.5 *μ*g/mL) for 24 h. All values are expressed as mean ± SEM of concentration of nitrite. One-way ANOVA was used followed by the Bonferroni posttest. ^#^vs. control group (DMSO 0.1%) (*p* < 0.05), ^∗^*p* < 0.05; ^∗∗^*p* < 0.01 vs. LPS group, *n* = 4/group.

Microglia plays a central role in neuroinflammation being responsible for the synthesis and release of a wide array of chemical mediators including cytokines such as TNF-*α*, IL-1*β*, IL-6, ROS, and NO. First, we investigated the effect of DEAC and its active principles (AMB, CM, and VA) on nitrite production of LPS-stimulated microglial cells. The addition of DEAC or its chemical constituents in increasing concentrations did not alter the basal levels of NO in BV-2 cells (data not shown). LPS increased NO release by approximately 13x (Figure 2) in microglial cells when compared to the untreated group (control group). This effect was significantly reduced by the pretreatment of cells with noncytotoxic concentrations of DEAC, AMB, or CM, reaching maximal reduction at 100 *μ*g/mL (nitrite: 6.1 ± 1.0 *μ*M, 4.4 ± 1.4 *μ*M, and 7.0 ± 1.5 *μ*M, respectively). The LPS-only groups released nitrite at the following concentrations: 13.0 ± 1.4 *μ*M, 16.7 ± 0.2 *μ*M, and 23.6 ± 2.8 *μ*M, respectively. AMB in particular showed a significant reduction in the NO production from 25 *μ*g/mL. The pretreatment of the BV-2 cells with VA did not significantly interfere in the NO production induced by LPS.

### 3.3. DEAC, CM, and AMB Regulate Cytokine Production in LPS-Stimulated Microglial Cells

**Figure 3:**
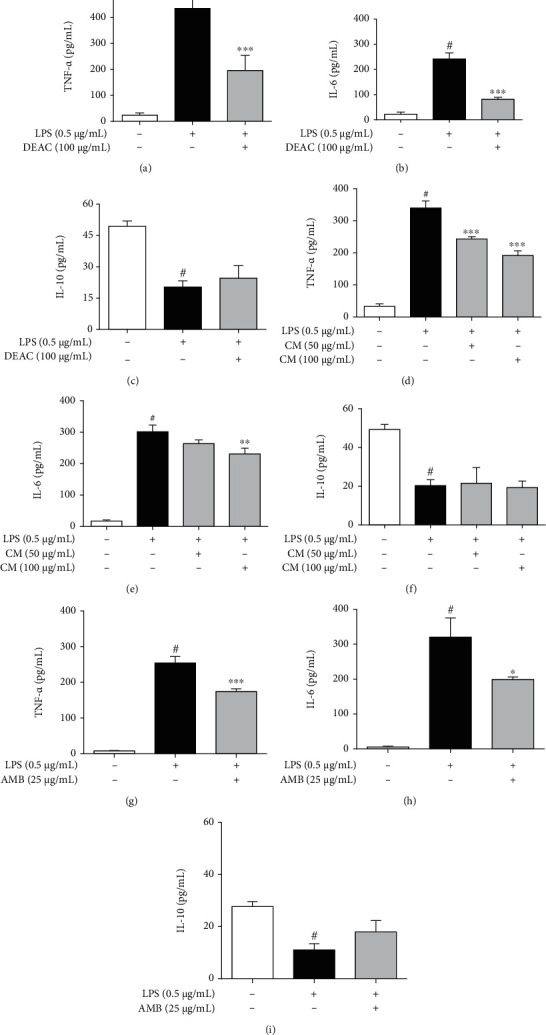
Effects of DEAC, CM, and AMB on inflammatory cytokine release in LPS-stimulated BV-2 cells. Cells were pretreated with DEAC (100 *μ*g/mL), CM (50 and 100 *μ*g/mL), and AMB (25 *μ*g/mL) for 1 h and activated with LPS (0.5 *μ*g/mL) for additional 24 h. The results are expressed as mean ± SEM. For statistical analysis, one-way ANOVA was used followed by the Bonferroni posttest. ^#^*p* < 0.05 vs. negative control (0.1% DMSO, drug vehicle); ^∗^*p* < 0.05, ^∗∗^*p* < 0.01, ^∗∗∗^*p* < 0.001 vs. LPS group, *n* = 4/group.

Considering the results obtained in the Griess assay, we chose the lowest anti-inflammatory concentration for each drug (DEAC, AMB, and CM) to evaluate their effects on the production of proinflammatory and anti-inflammatory cytokines (Figures 3(a)–3(i)). The addition of LPS for 24 h induced a significant increase in the TNF-*α*, IL-6, and IL-1*β* levels while IL-10 levels (anti-inflammatory cytokine) were reduced when compared to the untreated group. Pretreatment of cells with DEAC, AMB, or CM for 1 h before the addition of LPS significantly reduced the concentration of proinflammatory cytokines. On the other hand, the reduction of IL-10 induced by LPS on BV-2 cells was not significantly affected by the tested drugs, although DEAC and AMB increased its concentration by approximately 20.6% and 62.7%, respectively, compared to the LPS group.

### 3.4. DEAC, CM, AMB, and VA Have Free Radical Scavenging Activity: Hydroxyl Radical and Superoxide Anion

**Figure 4:**
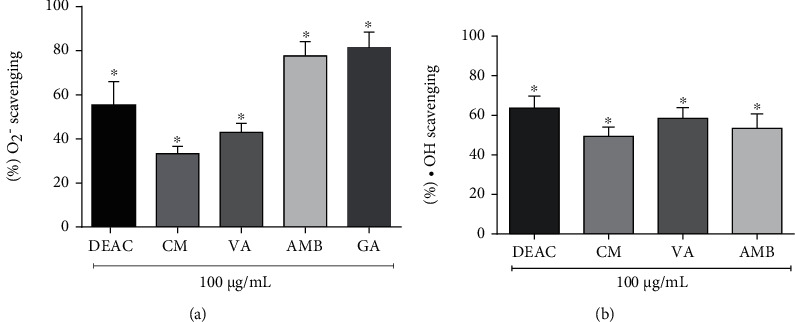
Antioxidant activity assay of DEAC, CM, VA, and AMB (100 *μ*g/mL). Scavenging effect on (a) superoxide anion and (b) hydroxyl radical. For statistical analysis, a one-sample *t*-test was used, ^∗^ vs. 100%.

An excessive amount of ROS produced by activated microglial cells can lead to cell damage and apoptosis. We investigated the possible oxygen free radical scavenging activity of DEAC and its active principles. DEAC, CM, VA, and AMB showed effective anion superoxide and hydroxyl radical scavenging activity (Figure 4). Superoxide anion scavenging activity of AMB (100 *μ*g/mL) was found to be 77.0 ± 12.3%, an effect comparable to gallic acid used as a reference compound (81.4 ± 7.1%). DEAC, CM, and VA exhibited scavenging activity of 55.2 ± 10.8%, 33.3 ± 3.3%, and 43.0 ± 4.0%, respectively. The scavenging effect of DEAC and its active principles on the hydroxyl radical decreased in the order of DEAC > VA > AMB > CM.

### 3.5. Effect of DEAC, AMB, and CM in LPS-Induced iNOS Expression in BV-2 Cells by Western Blot

**Figure 5:**
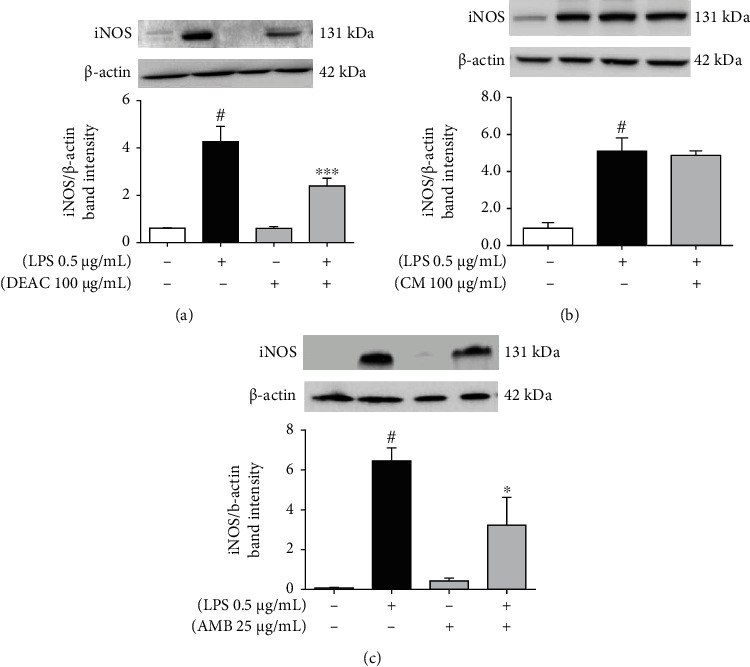
Effects of DEAC (a), CM (b), and AMB (c) on iNOS expression in LPS-stimulated BV-2 cells. Cells were pretreated with DEAC (100 *μ*g/mL), CM (50 and 100 *μ*g/mL), or AMB (25 *μ*g/mL) for 1 h and incubated with LPS (0.5 *μ*g/mL) for additional 24 h. Two-way ANOVA in graphs (a) and (c), and one-way ANOVA for graph (b), followed by Bonferroni posttest. ^#^*p* < 0.05 vs. negative control (0.1% DMSO, drug vehicle), ^∗∗^*p* < 0.01; ^∗∗∗^*p* < 0.001 vs. LPS group, *n* = 4/group.

Following the study, we investigated whether the suppression of NO production by DEAC, AMB, or CM was related to the expression of its synthase, iNOS. LPS induced a significant increase in the density of iNOS, but this effect was significantly reduced by the pretreatment with DEAC (100 *μ*g/mL) or AMB (25 *μ*g/mL) (Figure 5). On the other hand, the treatment of BV-2 cells with CM did not affect iNOS expression. In the absence of LPS, DEAC and its active principles did not change the expression of iNOS.

### 3.6. DEAC and CM Did Not Prevent the Reduction of Arginase Activity Induced by LPS in BV-2 Cells

The addition of LPS (0.5 *μ*g/mL) to BV-2 cells significantly reduced arginase activity, measured by the concentration of urea (50.5 ± 7.7 *μ*g/mL) when compared to the nontreated group (87.2 ± 11.6 *μ*g/mL). In the absence of LPS, only CM (100 *μ*g/mL: 10.3 ± 0.6 *μ*g/mL), but not DEAC (100 *μ*g/mL: 93.0 ± 12.0 *μ*g/mL), significantly reduced the concentration of urea. None of DEAC or CM was able to prevent the reduction of arginase activity when compared to the LPS group (LPS 0.5 *μ*g/mL), although the DEAC (100 *μ*g/mL: 58.2 ± 3.0 *μ*g/mL) showed a tendency to increase arginase activity by approximately 15.4%.

### 3.7. Effects of DEAC in LPS-Induced TLR-4/NF-*κ*B/ERK1/2 and JNK Pathways

**Figure 6:**
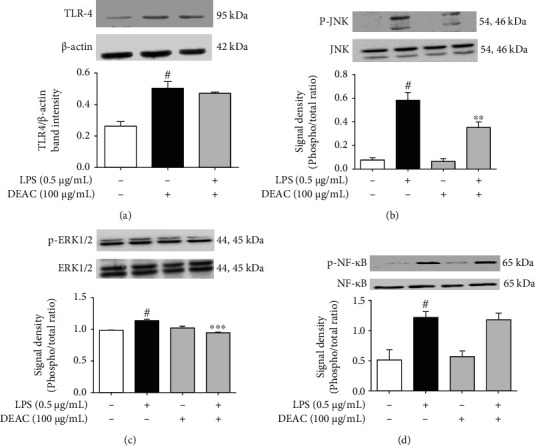
Effects of DEAC on TLR-4, p-NF-*κ*B, p-JNK, and p-ERK1/2 expression in LPS-stimulated BV-2 cells. Cells were pretreated with DEAC (100 *μ*g/mL) for 1 h and incubated with LPS (0.5 *μ*g/mL) for 1 more hour. The results are expressed as mean ± SEM. For statistical analysis, one-way ANOVA (a), two-way ANOVA (b–d), followed by Bonferroni posttest. ^#^*p* < 0.05 vs. negative control (0.1% DMSO, drug vehicle), ^∗∗^*p* < 0.01; ^∗∗∗^*p* < 0.001 vs. LPS group, *n* = 4/group.

LPS induces an inflammatory response by interacting with the TLR-4 receptor. We first investigated the effect of DEAC on the expression of this receptor in BV-2 cells, followed by the analysis of the phosphorylation of its downstream signaling proteins (NF-*κ*B and MAPKs). Western blotting results showed that the LPS exposure significantly increased the expression of TLR-4 in BV-2 cells when compared to the control group (not the treated group). However, DEAC pretreatment did not significantly prevent this increase (Figure 6(a)). As shown in Figures 6(b) and 6(c), LPS stimulation resulted in the phosphorylation of ERK1/2 and JNK, and DEAC pretreatment significantly decreased the phosphorylation of both MAPKs. Phosphorylation of NF-*κ*B was not changed by DEAC (Figure 6(d)).

## 4. Discussion

Evidence suggests that exacerbated neuroinflammation response in the central nervous system is decisively related to degenerative processes in several neurological diseases, including multiple sclerosis, Alzheimer's disease, and Parkinson's disease. Moreover, the literature has shown that microglial activation has a key role in the progression of those diseases [25–27]. Therefore, regulating neuroinflammation is a therapeutic goal. The present work sought to investigate the effects of standardized dried extract of *A. cearensis* (DEAC) and its chemical constituents (CM, AMB, and VA) in LPS-induced inflammation in microglial cells. When activated, these cells release several inflammatory and potentially neurotoxic mediators, including proteases, excitatory amino acids, cytokines, ROS, and reactive nitrogen species (RNS). Lipopolysaccharide, a bacterial endotoxin, is extensively used as an inflammatory stimulus for microglial activation [28]. This experimental model has been a useful tool to understand the role of neuroinflammation in the progression of neurological diseases. The production of inflammatory mediators after stimulation with LPS is the result of a sequence of reactions that begins at the binding of LPS to cell membrane receptors which activate a cascade of intracellular signaling pathways, resulting in the release of several mediators, including nitric oxide (NO). DEAC and its compounds, except VA, decreased nitrite production, elevated by the exposure to LPS, without significantly affecting cell viability, as assessed by the MTT test. Additionally, the plant extract and all chemical constituents evaluated (CM, AMB, and VA) showed antioxidant activity, acting as scavengers of hydroxyl and superoxide anion radicals. These results are consistent with previous studies developed by our laboratory which showed the anti-inflammatory and antioxidant effect of AMB and isokaempferide (flavonol) from *A. cearensis* in mesencephalic cells of rats exposed to the neurotoxin 6-hydroxydopamine (6-OHDA), human neutrophils, and/or CCl4-induced hepatotoxicity in rats [11, 18, 29].

Nitric oxide is associated with both beneficial and deleterious effects in humans. In the central nervous system (CNS), the iNOS upregulation and the accumulation of NO exacerbate the neuroinflammatory microenvironment, which contributes to neuronal cell death. The toxicity of NO is enhanced when it is combined with superoxide anion, generating peroxynitrite anion [30]. Decomposition of peroxynitrite results in the formation of some highly reactive species such as hydroxyl radicals [31]. Previous studies [32, 33] showed that higher hydroxyl radical levels and lower superoxide dismutase activity, an oxidoreductase responsible to dismutate the superoxide anion, seem to contribute to the onset and progression of neurodegenerative diseases, such as Parkinson's disease and Alzheimer's disease. Therefore, the reduction of nitrite concentration and the scavenging activity of hydroxyl and superoxide anion radicals exerted by DEAC and its active compounds suggest that DEAC's anti-inflammatory effects reduce the accumulation of reactive oxygen and nitrogen species (ROS/RNS) which play a major role in neuroinflammation-induced neuronal death [34]. Corroborating these data, Yeo et al. [35], describing the anti-inflammatory activity of a *Piper sarmentosum* extract in microglial cells, showed that the inhibition of NO production by the plant extract was associated with its free radical scavenging property.

Apart from NO, proinflammatory cytokines such as IL-1*β*, IL-6, and TNF-*α* are triggers of the inflammatory response and contribute to maintaining the high microglial activity that sustains the development of chronic inflammatory diseases [25]. These pathologies can be controlled by reducing the proinflammatory response and/or improving natural anti-inflammatory mechanisms. Thus, our study also investigated the effect of DEAC, CM, or AMB on the level of inflammatory and anti-inflammatory cytokines in LPS-stimulated BV-2 cells. Importantly, DEAC, CM, and AMB inhibited LPS-induced release of proinflammatory cytokines (TNF-*α* and IL-6), but we did not observe significant effects on the IL-10 (anti-inflammatory cytokine) reduction caused by LPS. Collectively, these data suggest that DEAC and its active principles CM and AMB may protect against neuronal death considering that IL-1*β* and TNF-*α* act synergistically as neurotoxic agents mediated at least in part by NO [36]. This hypothesis is corroborated by previous studies [15] where we described the anti-inflammatory and antioxidant activities of AMB from *A. cearensis* in mesencephalic cells of rats exposed to neurotoxin 6-OHDA.

NO synthesis can be limited by arginine availability and/or on the level of iNOS protein expression. During the NO synthesis, arginase-1 (expressed in microglial cells) competes with iNOS for L-arginine [37, 38]. Therefore, we investigated the effect of DEAC and its compounds on iNOS expression and arginase catalytic activity in LPS-stimulated BV-2 cells. We observed that LPS induced an increase in iNOS expression in BV-2 microglial cells, and pretreatment with DEAC or AMB decreased the expression of this enzyme. Meanwhile, the addition of CM in BV-2 cells did not affect the iNOS expression, and none of the test drugs improved the arginase activity when compared to the LPS-only group. Taken together, these results demonstrate that the inhibition of NO production by the extract of *A. cearensis* and AMB in microglial cells is related, at least in part, to their ability to reduce iNOS expression in microglial cells. Additional studies are necessary to describe how CM reduced the nitrite production in LPS-stimulated BV-2 cells without affecting iNOS expression. We hypothesize that it may act as an iNOS inhibitor.

**Figure 7:**
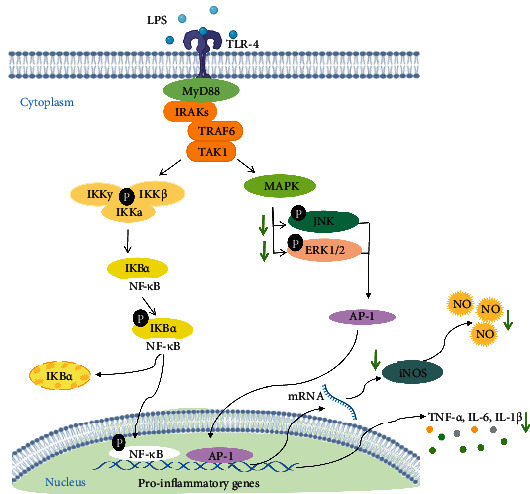
Proposed signaling mechanism for the effects of DEAC and chemical constituents (CM and AMB) in LPS-induced neuroinflammation in BV-2 cells. Activation of toll-like receptor 4 (TLR-4) with LPS leads to activation of the NF-*κ*B and MAPK pathways. The IKK complex phosphorylates IkB*α*, which leads to the degradation of IkB*α* and subsequent nuclear translocation of NF-*κ*B. At the same time, the MAPK pathway (JNK and ERK1/2) regulates the transcription of inflammatory mediators through the activation of the transcription factor AP-1. Activation of NF-*κ*B and AP-1 results in the expression of iNOS and the production of proinflammatory mediators. DEAC disrupts LPS-induced neuroinflammatory pathways, decreasing JNK and ERK1/2 signaling. DEAC and chemical constituents decrease the expression of iNOS, as well as the production of inflammatory mediators (NO and cytokines). IKK: I*κ*B kinase complex; I*κ*B*α*: kappa B inhibitor; P: phosphate; NF-*κ*B: nuclear kappa factor B; MAPK: mitogen-activated protein kinase; ERK: extracellular signal regulatory kinase; AP-1: activator protein 1.

The anti-inflammatory and antioxidant activities of DEAC described in the present study in microglial cells are corroborated by a previous study by Pereira et al. [13] which described the neuroprotective effect of an *A. cearensis* seed extract against neural damage induced by glutamate in cerebellar cells, which was associated to glial and neuronal preservation, and antioxidant activity. As DEAC's main bioactive markers CM and AMB showed a promising anti-inflammatory effect, we then further described the mechanisms underlying DEAC-mediated attenuation of inflammation in BV-2 cells. There are several published works describing *A. cearensis* or its active principles' anti-inflammatory or antioxidant activities [10, 11, 15, 17, 29, 39–41]. However, these studies are mainly confined to just showing their effects without further investigating the molecular mechanism underlying them. Therefore, the present study investigated the role of TLR-4, NF-*κ*B, and MAPK signaling pathways in the anti-inflammatory effect of DEAC in LPS-stimulated BV-2 cells. The binding of TLR-4 on the cell membrane by LPS in BV-2 cells activates various signaling cascades, including NF-*κ*B pathway via the MyD88–IRAK–TRAF6–TAK1 signaling complex leading to the activation of I*κ*B kinase complex (IKK). Activation of IKK includes the phosphorylation of NF-*κ*B which leads to its translocation to the nucleus. As a transcription factor, NF-*κ*B stimulates the synthesis of proinflammatory mediators including NO, IL-6, and TNF-*α* and the secretion of ROS. In addition to NF-*κ*B, LPS is a potent activator of the MAPK signaling pathways, which in turn modulate the activation of several transcription factors, including activator protein-1 (AP-1), STAT-1, and NF-*κ*B. AP-1 upregulates cytokine expression playing a key role in neuroinflammation [42–45]. In the present study, we found that DEAC treatment significantly inhibited LPS-stimulated phosphorylation of two MAP kinase pathways (JNK and ERK1/2) in microglial cells. However, DEAC did not interfere in the increased expression of TLR-4 or phosphorylation of NF-*κ*B induced by LPS (Figure 7).

Evidence demonstrates that excessive production of inflammatory mediators acts as inductors of MAPKs which in turn amplify and prolongs the inflammation process [45]. This vicious cycle leads to neuronal damage and death which is a hallmark of neurodegenerative diseases, such as Alzheimer's disease (AD) and Parkinson's disease (PD) [45]. Both JNK and ERK promote the phosphorylation of amyloid precursor protein that, when cleaved by *β*- and *γ*-secretases, yields the neurotoxic *β*-amyloid peptide which plays a key role in AD. In addition, studies demonstrated that TNF-*α*, a classical factor secreted from activated microglia, can induce a significant increase in the *α*-SNC secretion (a marker of PD) from both differentiated PC12 and SHSY5Y cells [35, 42]. Therefore, the inhibition of MAPK pathways by DEAC indicates that it may be a promising anti-inflammatory drug, useful for the treatment of neurodegenerative disorders.

The anti-inflammatory effect of DEAC is associated at least in part with the presence of AMB and CM in the plant extract, but the pharmacological importance of other chemical constituents from *A. cearensis* cannot be excluded. Moreover, the present work studied the effects of different drugs in an isolated microglial cell line; therefore, the anti-inflammatory activity observed may not fully represent an in vivo effect in reducing neuroinflammation. Because of this, future trials should be carried out using experimental animal models of neurodegenerative diseases to evaluate the effect of DEAC, AMB, and CM, investigating the anti-inflammatory, antioxidant, and consequent neuroprotective potential. The in vivo study will allow to know if these drugs have limitations associated to bioavailability and how they act in living system with complex interplay between the different cell types at different time points in the progression of neuroinflammatory diseases.

## 5. Conclusion

Taken together, our results demonstrate that DEAC and its active principles (AMB and CM) (100 *μ*g/mL) have free radical scavenging activity and inhibit the production of NO (up to 100 *μ*g/mL). At their concentrations that reduced at least 50% of NO production, they also reduced the release of the proinflammatory cytokines TNF-*α* and IL-6 as well as the expression of iNOS in LPS-stimulated BV-2 microglial cells (DEAC at 100 *μ*g/mL, CM at 50 and 100 *μ*g/mL, and AMB at 25 *μ*g/mL). These effects were not related to cytotoxicity and seem to occur through inhibition JNK and ERK1/2 MAPK pathways (DEAC at 100 *μ*g/mL). These data provide an experimental basis that the standardized dry extract of *A. cearensis* (cultivated species), CM, and AMB presents therapeutic potential for the treatment of inflammatory diseases where microglial activation plays a key role, such as neurodegenerative diseases.

## Data Availability

The data used to support the findings of this study are included within the article.

## Abbreviations

6-OHDA: 6-Hydroxydopamine
AD: Alzheimer's disease
AMB: Amburoside A
AP-1: Activator protein-1
BSA: Bovine serum albumin
CNS: Central nervous system
CM: Coumarin
DEAC: Dry extract from *Amburana cearensis*
DMSO: Dimethyl sulfoxide
EDTA: Ethylenediaminetetraacetic acid tetrasodium salt dihydrate
ERK: Extracellular signal-regulated kinase
FBS: Fetal bovine serum
FeCl_3_: Ferric chloride
H_2_O_2_: Hydrogen peroxide
H_3_PO_4_: Phosphoric acid
HPLC-PDA: High-performance liquid chromatography–photodiode array
HPX: Hypoxanthine
IFN-*γ*: Interferon-gamma
IKK: IkB kinase complex
IL: Interleukin
iNOS: Inducible nitric oxide synthase
JNK: C-Jun N-terminal kinase
L-Ascorbic acid: Vitamin C
LPS: Lipopolysaccharide
MAPKs: Mitogen-activated protein kinases
MTT: 3-(4,5-Dimethylthiazol-2-yl)-2,5-diphenyltetrazolium bromide
NBT: Nitrotetrazolium blue chloride
NEED: N-(1-Naphthyl)ethylenediamine dihydrochloride
NF-*κ*B: Nuclear factor kappa B
NO: Nitric oxide
PBS: Phosphate-buffered saline
PD: Parkinson's disease
RIPA: Radioimmunoprecipitation assay buffer
RNS: Reactive nitrogen species
ROS: Reactive oxygen species
SDS: Sodium dodecyl sulfate
TBA: 2-Thiobarbituric acid
TCA: Trichloroacetic acid
TLR-4: Toll-like receptor 4
TNF-ɑ: Tumor necrosis factor alpha
VA: Vanillic acid
XOD: Xanthine oxidase.
